# Maternally-Derived Antibodies Protect against Challenge with Highly Pathogenic Avian Influenza Virus of the H7N3 Subtype

**DOI:** 10.3390/vaccines7040163

**Published:** 2019-10-30

**Authors:** Stivalis Cardenas-Garcia, Lucas Ferreri, Zhimin Wan, Silvia Carnaccini, Ginger Geiger, Adebimpe O. Obadan, Charles L. Hofacre, Daniela Rajao, Daniel R. Perez

**Affiliations:** 1 Department of Population Health, University of Georgia, Poultry Diagnostic and Research Center, Athens, GA 30602, USA; lferreri@uga.edu (L.F.); 15952707826@163.com (Z.W.); scarnaccini@uga.edu (S.C.); imginger@uga.edu (G.G.); willowsgal@gmail.com (A.O.O.); daniela.rajao@uga.edu (D.R.); 2 Southern Poultry Research Group, Watkinsville, GA 30677, USA; clhofacre@thesprgroup.com

**Keywords:** maternally-derived antibodies, highly pathogenic avian influenza virus, H7N3, vaccination of chickens, protection

## Abstract

Vaccination of hens against influenza leads to the transfer of protective maternally-derived antibodies (MDA) to hatchlings. However, little is known about the transfer of H7N3 vaccine-induced MDA. Here, we evaluated transfer, duration, and protective effect of MDA in chickens against H7N3 HPAIV. To generate chickens with MDA (MDA (+)), 15-week-old White Leghorn hens were vaccinated and boosted twice with an inactivated H7N3 low pathogenic avian influenza virus vaccine, adjuvanted with Montanide ISA 71 VG. One week after the final boost, eggs were hatched. Eggs from non-vaccinated hens were hatched for chickens without MDA (MDA (−)). Both MDA (+) and MDA (−) hatchlings were monitored weekly for antibody levels. Anti-HA MDA were detected by hemagglutination inhibition assay mostly until day 7 post-hatch. However, anti-nucleoprotein MDA were still detected three weeks post-hatch. Three weeks post-hatch, chickens were challenged with 10^6^ EID_50_/bird of Mexican-origin H7N3 HPAIV. Interestingly, while 0% of the MDA (−) chickens survived the challenge, 95% of the MDA (+) chickens survived. Furthermore, virus shedding was significantly reduced by day 5 post-challenge in the MDA (+) group. In conclusion, MDA confers partial protection against mortality upon challenge with H7N3 HPAIV, as far as three weeks post-hatch, even in the absence of detectable anti-HA antibodies, and reduce virus shedding after challenge.

## 1. Introduction

Influenza A virus (IAV) infections of poultry have severe repercussions to the poultry industry. Infections with low pathogenic avian influenza virus (LPAIV) are commonly associated with reduced feed conversion rates, weight loss, decreased egg production, and secondary infections. Flocks infected with highly pathogenic avian influenza virus (HPAIV) usually show high mortality rates that can decimate an entire flock. Outbreaks of LPAI and HPAI cause important economic losses to the poultry industry due to expenses associated with culling and quarantine, emergency vaccination programs, and loss of consumer confidence, among other issues. Undoubtedly, the most important LPAIVs are of the H5 and H7 subtypes because of their potential to mutate into HPAIVs. Outbreaks in poultry caused by H5 and H7 subtype LPAIV and HPAIV strains are reportable to the World Animal Health Organization [1]. In the Americas, highly-pathogenic avian H7N3 viruses have been previously reported in Chile (2002) and Canada (2004 and 2007) [2,3,4]. In 2012, an H7N3 HPAIV was first reported in Mexico and became endemic with devastating consequences for the commercial poultry sector. Between June and August of 2012, 22.3 million chickens were culled [5,6]. Since then, Mexico has introduced intensive vaccination campaigns to control outbreaks occurring in different regions of the country [5], with only partial success as shown by continuing reports to the OIE [6]. H7N3 viruses isolated from Mexico have been further analyzed for protection in chickens and for their fitness in chickens and mallards. Variations in protection and fitness have been found between viruses isolated from different years as shown in recent reports [7,8].

Vaccination of poultry against influenza stimulates production of antibodies that confer variable protection against field strains. These antibodies can be vertically transferred from mothers to the hatching progeny and, therefore, are referred to as maternally-derived antibodies (MDA). The passive transfer of maternal immunity was initially described in chickens by Klemperer in 1893 (reviewed in [9]). There are three types of immunoglobulins identified in chickens: IgY (functional equivalent of the mammalian IgG), IgA, and IgM [10,11,12]. Serum IgY is transported from the hen’s blood stream into the oocyte (which becomes the egg yolk) while still in the ovarian follicle through a specific receptor that recognizes the Fc domain [13,14,15,16,17]. The IgA and IgM are secreted by the mucosa of the oviduct and incorporated in low amounts into the egg along with the secreted albumin during egg formation [18]. During embryo development, the IgY is transported from the egg yolk into the embryo’s systemic circulation through receptor-mediated transcytosis [19,20,21,22]. This transport has been documented as early as seven days into incubation [23] and increases over time, peaking towards the last couple of days before hatch and within the first few days after hatch [24]. The IgA and IgM are ingested by the embryo from the amniotic fluid and transported to the gut in very low amounts [15,18].

Several studies have been conducted to evaluate the half-life and protective capacity of MDAs against challenge with different pathogens, including avian-origin influenza viruses of various subtypes [25,26,27,28,29,30,31]. Hyper-immunization of hens through repeated vaccination is common in the poultry sector in order to maximize MDAs passed through the egg to the hatching progeny. Chicks frequently receive up to three weeks of protection from MDAs allowing their immune system to mature to a level capable of mounting an efficient active immune response if exposed to a harmful pathogen. Previous reports have highlighted the poor immune responses elicited by H7 viruses [32,33,34,35,36], which can result in poor protection of mice and chickens against lethal challenge [32,36]. These observations make it necessary to further explore alternatives that improve the stimulation of anti-HA antibodies as it has been shown previously [7,37,38,39]. The underlying mechanisms of such poor immunogenicity remain elusive; however, vaccines against H7 that produce negligible virus neutralizing responses continue to be widely used. More specific to this report, little is known about the role of anti-H7 MDAs in the prevention and control against H7 HPAIV strains in young chicks. In the present study, we assessed the antibody response in vaccinated hens with a LPAI H7N3 expressing a Mexican-lineage HA from 2015. In addition, we evaluated the transfer of these vaccine-induced antibodies to the offspring and their protection against a Mexican-lineage H7N3 HPAIV isolated in 2016.

## 2. Materials and Methods

### 2.1. Ethics Statement

All animal studies were approved and conducted in compliance with the regulations stated by the Institutional Animal Care and Use Committee (IACUC) of the University of Georgia (UGA). Vaccination studies were conducted under ABSL-2 conditions at the Poultry Diagnostic and Research Center (PDRC) at UGA. Challenge studies were carried out in an ABSL-3Ag containment facility at the Animal Health Research Center (AHRC) at UGA.

### 2.2. Cells, Eggs, and Chickens

Madin Darby canine kidney (MDCK) cells (ATCC^®^ CRL-2935™, Manassas, VA, USA) and 293T cells (ATCC^®^ CRL-3216™, Manassas, VA, USA) were used for reverse genetics and initial virus propagation. Specific pathogen-free (SPF) embryonated chicken eggs (ECEs) used for virus propagation and stock titration were obtained from Charles Rivers (Wilmington, NC, USA). Eight-week-old, commercial avian influenza-free White Leghorn poults were purchased from VALO BioMedia North America (Adel, IA, USA), and used in this study for the generation of MDA positive (MDA (+)) and MDA negative (MDA (−)) chickens. Poults were raised and housed at Southern Poultry Research Group facilities until the age of 15 weeks. All procedures performed on the hens took place at the same facility. Chickens were given food and water ad libitum for the duration of the experiment.

### 2.3. Viruses

The viruses were propagated in 10-day-old SPF ECEs as per standard protocols [1], and their identities were confirmed by sequencing analysis. The LPAIV rg_A/chicken/Mexico/CIP-102_RGSCG03/2015 (H7N3), used as vaccine strain for the hens, was rescued by reverse genetics [40] from synthesized gene segments with sequence homology to Mexican-lineage H7N3 viruses, and an HA gene segment from a virus isolated in Guanajuato, Mexico in 2015. The polybasic cleavage site (336PKDRKSRHRRTR347) from the HA gene segment was modified to generate a low-pathogenic, monobasic cleavage site (336PQIETR341). The 2016 Mexican HPAIV H7N3 derived-strain [rg_A/chicken/Mexico/CIP-102_RGSCG04/2016 (H7N3)] used for challenge was reconstituted by reverse genetics under BSL3-enhanced conditions using sequences provided by Dr. Alejandro Garcia (Avilab, Mexico), with the exception of PB2, which was derived from one of the Guatemalan wild bird-origin sequences described above. The rg_A/chicken/Mexico/CIP-102_RGSCG04/2016 (H7N3) virus had a typical HPAIV phenotype; it did not require trypsin to grow in tissue culture cells and displayed an intravenous pathogenicity index of 2.45 [1].

### 2.4. Vaccine Preparation

Allantoic fluid stock of the LPAIV rg_A/chicken/Mexico/CIP-102_RGSCG03/2015 (H7N3) strain was used to formulate an inactivated emulsified vaccine. To inactivate the viral stock, a 0.1 M solution of binary ethyleneimine (BEI) was prepared by mixing 2-bromoethylamine hydrobromide (Sigma-Aldrich, St. Louis, MO, USA) with a 0.175 N solution of sodium hydroxide (EMD Millipore Corporation, Burlington, NJ, USA). The solution was incubated at 37 °C for 1.5 h until pH dropped to 8.5. The solution was then filtered with a 0.22 μm filter (Corning, Corning, NY, USA) and mixed with the allantoic fluid stock to a final concentration of 0.01 M. Mix was incubated for 20 h at 37 °C, with continuous rocking to ensure homogeneous inactivation. Thereafter, the BEI was hydrolyzed with a 1 M sodium thiosulfate solution. BEI-inactivated stock pH was adjusted to 7.2 with 1 N hydrochloride solution, aliquoted and stored at −80 °C until needed. Three blind passages were performed in 10-day-old SPF ECEs to confirm inactivation. Vaccine emulsions were prepared by mixing Montanide ISA 71 VG (Lot. #36514P, SEPPIC, Paris, France) with BEI-inactivated viral stock (10^9.5^ EID_50_ /mL or 4096 hemagglutination units (HAU) before inactivation; 2048 HAU after inactivation) in a 73:27 ratio (*v*/*v*, Montanide: BEI-inactivated viral stock), according to SEPPIC specifications. Vaccine emulsions were stored at 4 °C until needed. In addition, testing for sterility (culture) and *Mycoplasma* spp. (PCR) contamination were performed.

### 2.5. Vaccination of Mothers and Generation of Hatchlings with and without MDA

White Leghorn hens (15-weeks old) were allocated into two different groups (*n* = 49 each) and kept off site at the Southern Poultry Research Group facilities. One group remained non-vaccinated. The second group of hens was vaccinated subcutaneously with 0.5 mL of the vaccine emulsion containing 5230 HAU/dose (described in Section 2.4). Vaccinated hens were boosted at 18- and at 22-weeks of age to increase antibody titers, administering 0.5 mL of vaccine emulsion per hen, subcutaneously. Serum samples were collected at 15, 18, 21, and 23 weeks of age to monitor the antibody response by ELISA against the nucleoprotein (NP) (IDEXX, Westbrook, ME, USA) and by the hemagglutination inhibition (HI) assay. One week after the final boost, fertile eggs were collected from both vaccinated and non-vaccinated hens. The embryos were transferred to the Poultry Diagnostic and Research Center at the University of Georgia and set to incubate until hatch. At hatch, a subset of chickens from both sets of eggs was tested for antibodies against NP and HA, by ELISA and HI, respectively, to confirm MDA status. Subsequently, chicks were allocated into two groups (*n* = 45/group, accounting for expected mortality due to age). The antibody response was monitored weekly by NP ELISA and HI. Terminal bleeding was performed, and serum collected from 10 chickens per group at days 7 and 14 after hatch, to assess the antibody levels. Survival bleeding was performed from 20 of the remaining chickens at day 20 after hatch.

### 2.6. Challenge of 21-Day-Old Chickens with or without MDAs

On day 21 after hatch, chickens (*n* = 20/group) were transferred to the ABSL-3Ag facility at the University of Georgia. The chickens were challenged intranasally with the HPAIV strain rg_A/chicken/Mexico/CIP-102_RGSCG04/2016 (H7N3), at a dose of 10^6^ EID_50_/bird. Chickens were monitored daily for 14 days after challenge to record clinical signs and mortality. Clinical signs were recorded on 4 different categories, including level of activity, physical appearance, respiratory signs, and other clinical signs. Activity and physical appearance were assigned scores from 0 = normal to 3 = severe and graphed to compare between groups. Terminal bleeding was performed at the end of the experimental period, 14 days post-challenge (dpc), and sera were analyzed for antibody titers by HI assay as described below. Tracheal and cloacal swab samples were collected on days 2 and 5 after challenge.

### 2.7. Virus Shedding after Challenge

RNA was extracted from the swab material and the challenge virus stock using the Ambion’s MagMAX™-96 AI/ND Viral RNA Isolation Kit (ThermoFisher Scientific, Waltham, MA, USA). Virus shedding was determined by RT-qPCR using qScript™ XLT One-Step RT-qPCR ToughMix^®^, QuantaBio master mix (VWR, Radnor, PA, USA) using the Applied Biosystems 7300 instrument. A standard curve was prepared using RNA extracted from the challenge virus stock. Virus shedding titers are expressed as EID_50_ /mL equivalents plus minus the standard deviation (SD).

### 2.8. Hemagglutination Inhibition (HI) Assay

Whole blood was collected, and the sera were separated by centrifugation at 1000× *g* for 15 min at room temperature. Sera was then pre-absorbed with a 10% suspension of chicken red blood cells (chRBC) as follows: 50 µL of serum were mixed with 100 µL of 1× phosphate buffered saline (PBS) and 50 µL of chRBC 10% and incubated at room temperature for 30 min, shaking samples every 10 min. Erythrocytes were allowed to settle down at 4 °C for 2 h. The treated sera were then collected into new sample tubes and brought to a final dilution of 1:10. The HI assay was performed in V-bottomed microtiter plates, using four hemagglutination units (HAU) of viral antigen per 25 µL, as recommended by the OIE [1]. Briefly, two-fold serial dilutions were prepared by mixing 25 µL of 10-fold-diluted sera with 25 µL of 1× PBS. Next, 4 HAU (in 25 µL) of rg_A/chicken/Mexico/CIP-102_RGSCG03/2015 (H7N3) (vaccine virus) were added to each sera dilution and incubated for 15 min at room temperature. Finally, 50 µL of a 0.5% suspension of chRBC were added to serum-antigen mixes and incubated for 45 min at room temperature before reading the results. The highest dilution that showed complete hemagglutination inhibition was considered the HI titer for such sample. HI titers expressed as dilution values were plotted using Prism v8.0.2 (GraphPad Software, San Diego, CA, USA). The limit of detection was at dilution 1/10, and samples with undetectable titers were assigned a dilution value of 1/8 for statistical purposes.

### 2.9. Statistical Analysis

Mean HI data from vaccinated mothers and MDA (+) progeny were analyzed by one-way ANOVA followed by post hock Holm–Sidak’s multiple comparison test to determine differences between time points. Virus shedding data was analyzed by two-way ANOVA to compare virus shedding levels between the two groups at two different time points. Survival curves were analyzed using the log-rank test. The level of significance for all the analysis was considered at *p* < 0.05. All the analyses were performed using Prism v8.0.2.

## 3. Results

### 3.1. Antibody Response against the H7N3 Influenza Virus in Vaccinated Hens

**Figure 1:**
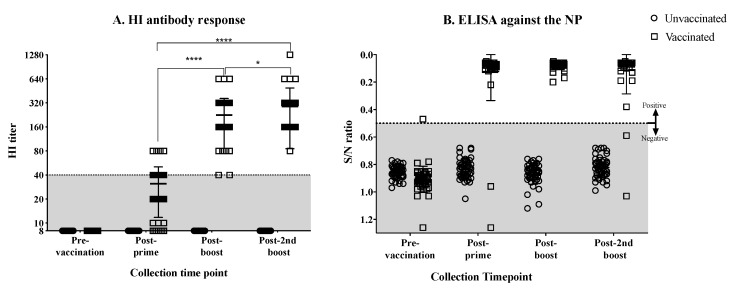
Antibody responses in hens vaccinated and boosted with the BEI-inactivated rg_A/chicken/Mexico/CIP-102_RGSCG03/2015 (H7N3) vaccine. (**A**) Anti-HA antibody titers measured by HI assay from serum collected from vaccinated and unvaccinated hens before and after prime and boost vaccinations. (**B**) Anti-NP antibody titers measured by ELISA from serum collected from vaccinated and unvaccinated hens. Anti-HA antibody titers increased considerably after the first boost as shown by the HI titers (A). No changes were observed on anti-NP antibody titers after boost (B). Titers from individual hens are shown per group and time point; bars indicate the mean titer ± SD. HI titers lower than 40 are considered to be under the predictive level of protection in adult humans (shaded area). Statistically significant differences between groups are denoted with stars (*). * *p* < 0.05, **** *p* < 0.0001. S/N = sample to negative control ratio. SD = standard deviation of the mean.

Hens (15-weeks old, *n* = 49) were vaccinated and boosted twice with emulsified, BEI-inactivated, LPAI rg_A/chicken/Mexico/CIP-102_RGSCG03/2015 (H7N3) vaccine virus. Most of the hens showed low HI responses after priming. The majority of the hens showed HI titers of 40, few had titers of 80 (Figure 1A). After the first boost, the mean HI response increased significantly (*p* < 0.0001), displaying HI titers ranging from 40 to 640, with the majority of the hens presenting titers of 160 and 320 (Figure 1A). By the second boost, the mean HI titer increased slightly more and was significantly different than the mean titer after priming and after the first boost (*p* < 0.0001, *p* < 0.05, respectively). After the second boost, HI titers ranged from 80 to 640, with one hen showing a titer of 1028 (Figure 1A). The antibody response was simultaneously monitored by measuring anti-NP antibodies by ELISA. Results showed less variation in the NP antibody titers (Figure 1B). As expected, the non-vaccinated hens remained negative throughout this phase of the study. After the second vaccine boost, fertile eggs were collected from vaccinated and non-vaccinated control hens and were set to hatch.

### 3.2. Significant Drop in the Hatchlings’ HI Titers by the Second Week Post-Hatch

**Figure 2:**
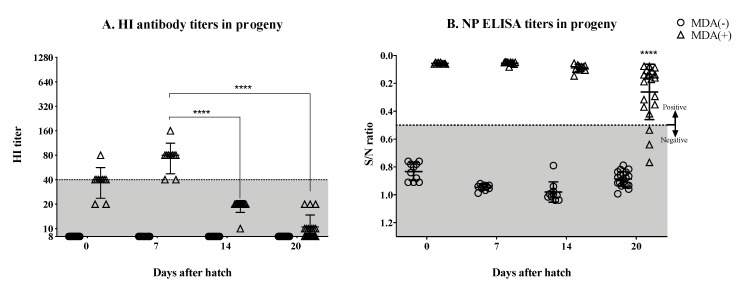
Monitoring MDA after hatch. Hatchlings originated from unvaccinated (MDA (−)) and vaccinated (MDA (+)) hens were monitored for the presence of maternally-derived antibodies every week starting at hatch. (**A**) Anti-HA antibodies measured by HI assay at hatch, 7, 14, and 20 days post-hatch. (**B**) Anti-NP antibodies measure by ELISA at hatch, 7, 14, and 20 days post-hatch. Detection of anti-HA antibodies drastically decreased after the first week post-hatch (A) while the NP antibodies were still detectable at least until day 20 post-hatch (B). Titers from individual chickens are shown per group and time point; bars indicate the mean titer ± SD. HI titers lower than 40 are considered to be under the predictive limit of protection for adult humans (shaded area). Statistically significant differences between groups are denoted with stars (*). **** *p* < 0.0001. S/N = sample to negative control ratio. SD = standard deviation of the mean.

Upon hatch, a subset of hatchlings was sacrificed and bled out to confirm the status of the MDA response (Figure 2A,B). Hatchlings from vaccinated hens showed evidence of MDA titers against the HA, with HI titers ranging from above limit of detection to 80, with most chicks at the 40 mark. These same chicks showed marked NP ELISA titers well above the cut off mark, strongly indicating proper transfer of MDAs to hatchlings, as expected. In contrast, and also as expected, hatchlings from non-vaccinated hens had HI titers below the limit of detection and were negative by NP ELISA. The remaining hatchlings were monitored weekly through HI and NP ELISA, respectively, to study the kinetics of the MDAs (Figure 2A,B). Seven days after hatch, the HI titers in the MDA (+) group increased slightly compared to the same group at day 0 with HI titers from 40 to 160 (one bird), with the majority of the chicks at the HI = 80 mark. However, by day 14 after hatch, the MDA HI titers dropped significantly to HI = 20 or lower (*p* < 0.0001). HI titers continued to drop by day 20 after hatch (*p* < 0.0001), where 10 out of 20 chickens had undetectable HI titers, and seven and three chickens had HI titers of 10 and 20, respectively (Figure 2A). However, our limit of detection was 10 and the possibility of there being weaker responders with titers lower than 10 cannot be discounted. In contrast, MDA NP ELISA titers were still detected in these chickens by day 14 after hatch, and in most of them by day 20 after hatch. The mean NP ELISA titer by day 20 after hatch had dropped significantly compared to days 7 (*p* < 0.0001) and 14 (*p* = 0.001) after hatch (Figure 2B).

### 3.3. MDA Ameliorate the Clinical Outcome and Partially Protect against Mortality after Challenge with HPAIV

**Figure 3:**
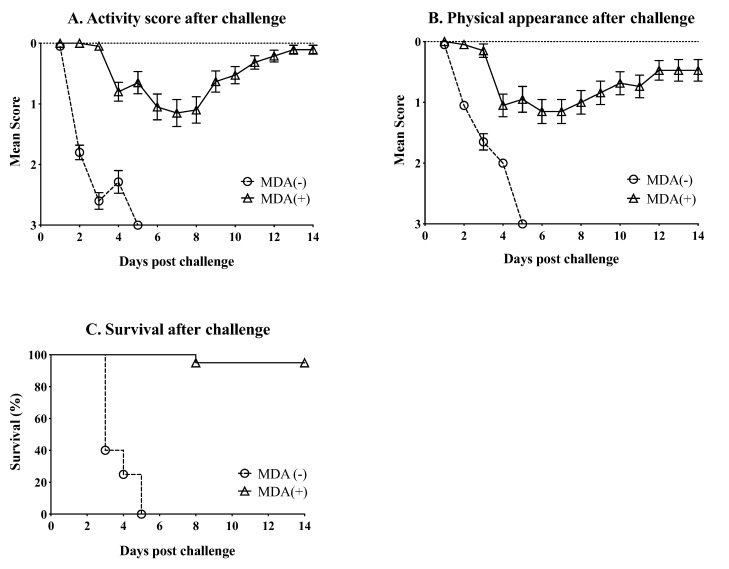
Protection conferred by MDA upon challenge. 21 days-old chickens (MDA (+) and MDA (−)) were challenged with 10^6^ EID_50_/chicken of rg_A/chicken/Mexico/CIP-102_RGSCG04/2016 (H7N3). Mortality and clinical signs were monitored for 14 days after challenge. (**A**) Level of activity; 0 = normal, 3 = severe; scores expressed as the group mean ± SEM. (**B**) Physical appearance; 0 = normal, 3 = severe; scores expressed as the group mean ± SEM. (**C**) Survival after challenge; survival represented as percentage from the total number of chickens allocated per group. Bars represent mean titers ± SD. Statistically significant differences between groups are denoted with stars (*) when appropriate. SD = standard deviation of the mean.

On day 21 after hatch, chickens from the MDA (+) and MDA (−) groups (*n* = 20/group) were challenged with a lethal dose of 10^6^ EID_50_/bird of the H7N3 HPAIV rg_A/chicken/Mexico/CIP-102_RGSCG04/2016 (H7N3) strain. After challenge, chickens were monitored daily for 14 days for clinical signs of infection and mortality. Chickens in the MDA (−) group showed more sudden and severe clinical disease after challenge compared with chickens in the MDA (+) group (Figure 3A,B). MDA (−) chickens showed reduced levels of activity and severe clinical appearance. MDA (−) chickens rapidly developed conjunctivitis and swelling and edema around the eyes, ruffled feathers, and severe depression. However, many chickens in the MDA (−) group showed sudden death without noticeable clinical signs after challenge. Few MDA (−) cases presented with tremors, incoordination and paralysis in otherwise healthy-looking chickens. Most of the MDA (−) chickens (*n* = 12) had succumbed to the challenge virus by 2 dpc, and by 5 dpc, all of them had died (Figure 3C). In contrast, MDA (+) chickens were within normal parameters within the first 3 dpc. By 4 dpc, MDA (+) chickens showed reduced activity levels and presented with clinical signs of disease, which were milder compared to those observed in MDA (−)/challenge chickens (Figure 3A,B). The clinical signs in MDA (+) chickens remained about the same for the next 5 days. By 9 dpc, MDA (+) chickens started to recover. By 14 dpc, MDA (+) chickens had mostly recovered from the challenge (Figure 3A,B). More importantly, most MDA (+) chickens survived the challenge (19 out of 20). A single MDA (+) chicken died by 8 dpc (Figure 3C).

### 3.4. MDAs Lead to Reduced Virus Shedding after Challenge

**Figure 4:**
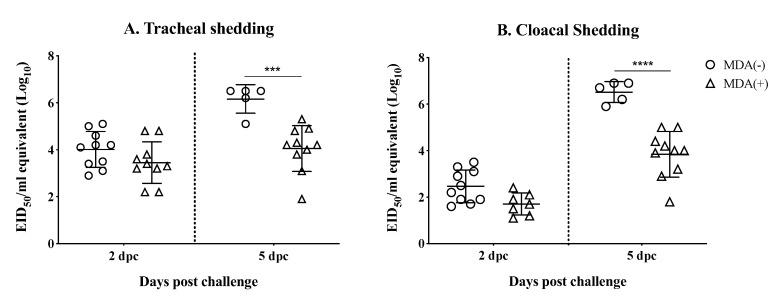
Virus shedding after challenge. 21 days-old chickens (MDA (+) and MDA (−)) were challenged with 10^6^ EID_50_/chicken of rg_A/chicken/Mexico/CIP-102_RGSCG04/2016 (H7N3). Tracheal and cloacal swab samples were collected 3 and 5 dpc for virus shedding determination. (**A**) Tracheal virus shedding. (**B**) Cloacal virus shedding. Titers from individual chickens are shown; bars represent mean titers ± SD. Statistically significant differences between groups are denoted with stars (*) when appropriate. *** *p* < 0.001, **** *p* < 0.0001. SD = standard deviation of the mean.

Viral RNA was extracted from tracheal and cloacal swab samples collected at 2 and 5 dpc and virus shedding was inferred from RT-qPCR data (Figure 4A,B). Chickens from the MDA (+) group shed slightly (*p* > 0.05) less virus than the MDA (−) chickens by 2 dpc from the trachea (10^3.45^ ± 0.886 vs 10^4.01^ ± 0.767, respectively) (Figure 4A) and from the cloaca (10^2.46^ ± 0.7 vs 10^1.7^ ± 0.473, respectively) (Figure 4B. However, this difference became more significant by 5 dpc for both tracheal shedding (MDA (+) group 10^4.05^ ± 0.974 vs. MDA (−) 10^6.16^ ± 1.127, *p* < 0.001) (Figure 4A) and cloacal shedding (MDA (+) group 10^3.84^ ± 0.98 vs. MDA (−) 10^6.52^ ± 0.449, *p* < 0.0001) (Figure 4B). Overall, these results suggest that MDAs help decrease shedding of the challenge virus.

## 4. Discussion

Where vaccines are used, vaccination of hens against avian influenza is typically practiced in the field to provide the hatchlings with protective antibodies within the first three weeks post-hatch. Such practice, however, is performed with the notion that subsequent vaccination of the hatchlings will occur 2–3 weeks post-hatch as MDAs tend to block vaccine antigens. Pathogen-specific MDAs in chickens are mostly represented by IgY and transferred to the embryo through the egg yolk [31]. MDA transfer from the hens to the offspring, is proportional to the level of antibodies present in the hen’s serum. These antibodies can be detected in the hatchling’s serum right after hatch and continue to be absorbed during the first day of age. It has been reported that the level of MDA represents approximately 30% of the antibody levels present in the mothers [31]. MDAs against H5 and H9 have been previously studied in chickens [26,27,29,30,41]. In contrast, little is known about MDA against H7N3 viruses. This is particularly important since the H7 subtype HA is notorious for its poor immunogenicity [33,42,43]. Here, we evaluated the immunogenicity of a Mexican-like LPAI H7N3 vaccine in hens and its capacity to induce and transfer protective antibodies to the offspring. In our study, we utilized an inactivated, adjuvated, H7N3 virus vaccine derived from a Mexican-lineage avian-origin H7N3 virus to immunize hens and resembles a common practice in the field. The results showed stimulation of HI antibody responses in hens following a prime and double boost vaccination regime. The HI titers against the H7 strain in hens were clearly discernible particularly after boost vaccinations. A previous report in three-week-old chickens that were vaccinated with various HA units of an H7N7 inactivated vaccine showed lower mean HI titers than those from our study. However, the highest vaccine dose used in the previous study was a least 10 times lower than the dose used in our study [44]. We performed HI assays from a subset of progeny chickens that were sacrificed at hatch from the vaccinated hens showing a mean HI titer with the minimum predictive protective value (HI ~40) (Figure 2A). By day 7 after hatch, chickens from the MDA (+) group showed HI titers to well within predictive protective value (HI ≥ 40). The transfer to hatchlings of vaccine-induced anti-H7 antibodies were approximately 3 log2 lower than the serum HI titers detected in the mothers, consistent with previous observations [26]. Such titers quickly decrease below predictive protective values by day 14 post-hatch (and remain low by day 20 post-hatch). Previous studies have reported a decline in MDA titers that varies depending on the pathogen and the strain. Studies performed using inactivated virus vaccines against H5 subtype HPAIV in broiler chickens, showed significant decrease of MDA-derived HI titers against the homologous H5 HA antigen between days 3 and 14 after hatch [26,29]. Likewise, a progeny from a broiler flock vaccinated with an H9N2 vaccine virus showed dramatic decrease of MDA titers (determined by ELISA) by day 5 after hatch [30]. Altogether, the previous studies and our current report suggest that the decay of MDAs is likely influenced by a combination of factors including, but not limited to, the vaccine antigen, the vaccine strategy and platform and the chicken breed. Further studies will be needed to further elucidate these features.

HI data has been used as reliable indicator of MDA status. However, the results from the present study and field observations, suggest that HI data is not good enough to establish MDA status of the flock beyond the first week of age. As a complement to the HI data, we detected NP antibodies after vaccination and after hatching. Although the NP antibodies are not considered to have protective value, commercial NP ELISAs are readily available and widely used for detection of avian influenza exposure. Since we used a whole virus inactivated vaccine, detection of NP antibodies after vaccination and hatching were not unexpected and highlights that antibodies not only against HA but also other viral antigens were stimulated in the mothers and consequently transferred to the offspring. While the HI titers drastically decreased by day 14 after hatch, the anti-NP antibodies were clearly detectable at least until day 20 post hatch. Our understanding is that NP is the second most abundant viral protein (~1000/virion) and antibodies to the NP represent about 10% of the relative response to influenza viruses [45]. However, at this stage it is not clear why antibodies to NP would remain in circulation longer than those with HI activity. Could the poor detection of anti-H7 antibodies be related to the assay employed (HI) in this study? It is well established that HI assays do not capture the entire spectrum of anti-HA antibodies. It remains to be assessed whether the use of an ELISA specific to the H7-HA in question would result in discernible anti-HA antibodies. Since H7 vaccines tend to show poor immunogenicity, our results suggest that HI data should be combined with other analytical methods (such as the NP ELISA if a whole inactivated vaccine is used) to better assess the MDA status of a flock and better predict protection and/or potential interference with vaccine efficacy. In adult humans, HI titers ≥ 40 have been considered to be the predictive limit of protection for seasonal influenza viruses [46,47,48]. However, in the case of avian-origin AIV antigens of poor immunogenicity HI values that correlate with protection would probably need to be redefined.

The underlaying mechanism of protection against HPAIV H7N3 observed in our study, in the absence of detectable HI antibodies remains ill-defined. Remarkable protection against disease and death was achieved in chickens with MDAs challenged with an aggressive dose of H7N3 HPAIV at 21 days post-hatch. Although all MDA (+) chickens showed signs of disease after challenge, only one of them died (out 20). This is in stark contrast to the MDA (−) group in which all the chickens succumbed to the infection by 5 day (the majority by 2 dpc). The level of protection observed corresponded also with lower virus shedding after challenge in the MDA (+) group compared to the MDA (−) group at 2 and 5 dpc in both tracheal and cloacal swabs. Such observation is in agreement with previous findings using a H5 vaccine [26]. We must note that in this study, the HAs of H7 vaccine and H7 challenge virus share 97.5% amino acid sequence identity, therefore, the protective responses are not a direct result of a homologous challenge in the strict sense. More importantly, protection was not directly correlated to HI titers since those were ≤20 at the time of challenge. This is in contrast with the previous H5 virus vaccine study that showed poor survival after challenge with an H5N1 HPAIV in two-week-old chickens that had MDA-derived HI titers <64 at time of challenge [26]. Several potential factors may have contributed to the protective responses observed in our study. Previous studies have shown the capacity of anti-NP antibodies at inducing complement-mediated lysis of influenza-infected cells in vitro [49]. In a different study, NP-immunized mice showed the potential for anti-NP antibodies to ameliorate clinical disease and help with viral clearance after challenge with H1N1 influenza virus [50]. Anti-NP serum given to unimmunized mice, was also able to achieve the same effect as in the donor mice against challenge [50]. Alternatively, antibodies directed to the HA, but without HI activity, or directed to the NA could contribute to protection. Anti-HA antibodies directed to the stalk are not as protective as those directed to the globular head [51]. However, a challenge study performed in a volunteer human cohort showed that subjects with anti-stalk antibodies were less likely to shed virus [52]. Likewise, anti-NA antibodies do not prevent infection but can inhibit neuraminidase activity that affect virus budding off the cell [53]. A recent study performed in mice showed cross protection of anti-NA antibodies against challenge with H3N2, H1N1, and HPAI H5N1 viruses [53]. Altogether, the protective mechanism induced by the anti-H7N3 MDA in chickens is most likely multifactorial and further studies will be needed, which are beyond the scope of the present report.

Vaccine studies performed with avian-origin H7N9 viruses isolated from humans have shown the poor immunogenicity of the vaccine. H7N9 vaccine candidates for potential human use must be adjuvanted in order to induce serum responses with predictive protective value [32,34]. It has been suggested that the H7 subtype HA of H7N9 viruses has lower numbers of T cell epitopes and a particular epitope that downregulates stimulation of T-helper cells and upregulates activation of regulatory T cells, both of which would lead to decreased cellular and antibody responses against the virus in humans [33,42]. However, such observations have not been proven to occur in chickens. Thus, the underlying mechanisms that affect the immunogenicity of avian-origin H7 subtype influenza viruses in chickens remains to be elucidated.

In summary, vaccine-induced MDAs can protect chickens against challenge with a H7N3 HPAIV strain even in the absence of discernable HI titers. The lack of detectable HI titers after hatch or vaccination is a common problem in the field that makes it difficult to field veterinarians to predict protection and/or to decide when to vaccinate since MDAs are known to interfere with live and inactivated vaccines. Complementary diagnostic methods, such as the NP ELISA used here or optimized enzyme-linked lectin assays to detect NA antibodies should be implemented. Further studies are warranted to better correlate the predictive protective value of complementary diagnostic methods.
